# COXIBs and 2,5-dimethylcelecoxib counteract the hyperactivated Wnt/β-catenin pathway and COX-2/PGE2/EP4 signaling in glioblastoma cells

**DOI:** 10.1186/s12885-021-08164-1

**Published:** 2021-05-03

**Authors:** Aleksandra Majchrzak-Celińska, Julia O. Misiorek, Nastassia Kruhlenia, Lukasz Przybyl, Robert Kleszcz, Katarzyna Rolle, Violetta Krajka-Kuźniak

**Affiliations:** 1 Department of Pharmaceutical Biochemistry, Poznan University of Medical Sciences, Poznań, Poland; 2 Department of Molecular Neurooncology, Institute of Bioorganic Chemistry Polish Academy of Sciences, Poznań, Poland; 3 Laboratory of Mammalian Model Organisms, Institute of Bioorganic Chemistry Polish Academy of Sciences, Poznań, Poland

**Keywords:** GBM, COXIBs, 2,5-dimethylcelecoxib, Wnt/β-catenin signaling pathway

## Abstract

**Background:**

Glioblastoma (GBM) is the deadliest and the most common primary brain tumor in adults. The invasiveness and proliferation of GBM cells can be decreased through the inhibition of Wnt/β-catenin pathway. In this regard, celecoxib is a promising agent, but other COXIBs and 2,5-dimethylcelecoxib (2,5-DMC) await elucidation. Thus, the aim of this study was to analyze the impact of celecoxib, 2,5-DMC, etori-, rofe-, and valdecoxib on GBM cell viability and the activity of Wnt/β-catenin pathway. In addition, the combination of the compounds with temozolomide (TMZ) was also evaluated. Cell cycle distribution and apoptosis, *MGMT* methylation level, COX-2 and PGE2 EP4 protein levels were also determined in order to better understand the molecular mechanisms exerted by these compounds and to find out which of them can serve best in GBM therapy.

**Methods:**

Celecoxib, 2,5-DMC, etori-, rofe- and valdecoxib were evaluated using three commercially available and two patient-derived GBM cell lines. Cell viability was analyzed using MTT assay, whereas alterations in *MGMT* methylation level were determined using MS-HRM method. The impact of COXIBs, in the presence and absence of TMZ, on Wnt pathway was measured on the basis of the expression of β-catenin target genes. Cell cycle distribution and apoptosis analysis were performed using flow cytometry. COX-2 and PGE2 EP4 receptor expression were evaluated using Western blot analysis.

**Results:**

Wnt/β-catenin pathway was attenuated by COXIBs and 2,5-DMC irrespective of the COX-2 expression profile of the treated cells, their *MGMT* methylation status, or radio/chemoresistance. Celecoxib and 2,5-DMC were the most cytotoxic. Cell cycle distribution was altered, and apoptosis was induced after the treatment with celecoxib, 2,5-DMC, etori- and valdecoxib in T98G cell line. COXIBs and 2,5-DMC did not influence *MGMT* methylation status, but inhibited COX-2/PGE2/EP4 pathway.

**Conclusions:**

Not only celecoxib, but also 2,5-DMC, etori-, rofe- and valdecoxib should be further investigated as potential good anti-GBM therapeutics.

**Supplementary Information:**

The online version contains supplementary material available at 10.1186/s12885-021-08164-1.

## Background

GBM represents the most aggressive and the most common primary brain tumor in adults. It is highly heterogeneous and driven by diverse genetic, epigenetic, and developmental programs [1]. Despite surgical resection followed by radio- and chemotherapy, GBM tends to recur and only fewer than 5% of patients survive 5 years after diagnosis [2]. Novel therapies, drug targets, and drug combinations are needed to prolong GBM patients lifespan [3–6].

One of the most critical oncogenic drivers of GBM is the Wnt/β-catenin pathway [7]. As we and others have previously shown, hyperactivation of this signaling pathway is mainly caused by promoter methylation of its inhibitors [8–10]. Without the protective role of its natural antagonists, Wnt pathway is hyperactivated, contributing to the maintenance of GBM cells stemness, invasiveness, and angiogenesis, as well as chemio- and radioresistance [7]. Therefore, Wnt/β-catenin pathway is postulated as a promising target in GBM therapy. Moreover, inflammation is crucial for GBM progression. Cyclooxygenase 2 (COX-2) is expressed in GBM cells, including glioma stem cells, and plays a key role in the production of the bioactive lipid, prostaglandin E2 (PGE2). The latter increases the activation of TCF/LEF transcription factors, activates the Wnt pathway and promotes GBM cells proliferation [11]. Moreover, PGE2 increases the nuclear accumulation of β-catenin, needed for Wnt pathway activation [12]. Therefore, COX-2/PGE2 pathway has also been suggested as a potential anti-glioma target [13, 14]. PGE2 induces also DNMT3B (DNA methyltransferase 3B) expression and activity, which in turn can potentially result in higher level of *MGMT* (*the O*^*6*^*-methylguanine-DNA methyltransferase*) promoter methylation. This is beneficial in the context of temozolomide (TMZ) response [15].

Several analogs of selective COX-2 inhibitors (COXIBs) exist [16]. Celecoxib, besides its anti-inflammatory effects, has been reported to have anti-neoplastic activity against several malignancies, including GBM. A retrospective clinical study in recurrent GBM showed six months progression-free survival of 43% of patients treated with low-dose TMZ plus celecoxib, as opposed to the 21% treated with standard TMZ maintenance therapy [17]. Celecoxib was also included as part of ‘CUSP9’ treatment protocol, as one of nine drugs inhibiting growth-enhancing pathways of GBM [18]. Evidence shows that celecoxib acts not only through COX-2 inhibition, but also via the Wnt/β-catenin pathway, and the effect is exerted even on glioma stem cells [19]. Interestingly, celecoxib analog, 2,5-dimethylcelecoxib (2,5-DMC), lacking COX-2 inhibitory activity, and being superior to celecoxib for antitumor purposes, was also reported to inhibit the Wnt pathway [20]. These promising findings refer to intestinal cancer cells. In contrast, the impact of 2,5-DMC on GBM cells needs to be further elucidated, especially in GBM patient-derived primary cell lines. Moreover, little is known about the effect of other COXIBs, such as etori-, rofe- and valdecoxib on the Wnt/β-catenin pathway and GBM cells viability. The ability of COXIBs to induce cell cycle arrest and apoptosis in GBM cells as well as alter *MGMT* methylation status also requires further investigation.

Thus, the aim of this study was to analyze the impact of celecoxib, 2,5-DMC, etori-, rofe-, and valdecoxib on GBM cell viability and the activity of Wnt/β-catenin pathway. In addition, the combination of the compounds with TMZ was also evaluated. Cell cycle distribution and apoptosis, *MGMT* methylation level, COX-2 and PGE2 EP4 protein levels were also determined in order to better understand the molecular mechanisms exerted by these compounds and to find out which of them can serve best in GBM therapy.

## Methods

### GBM cell lines and culture

**Fig. 1:**
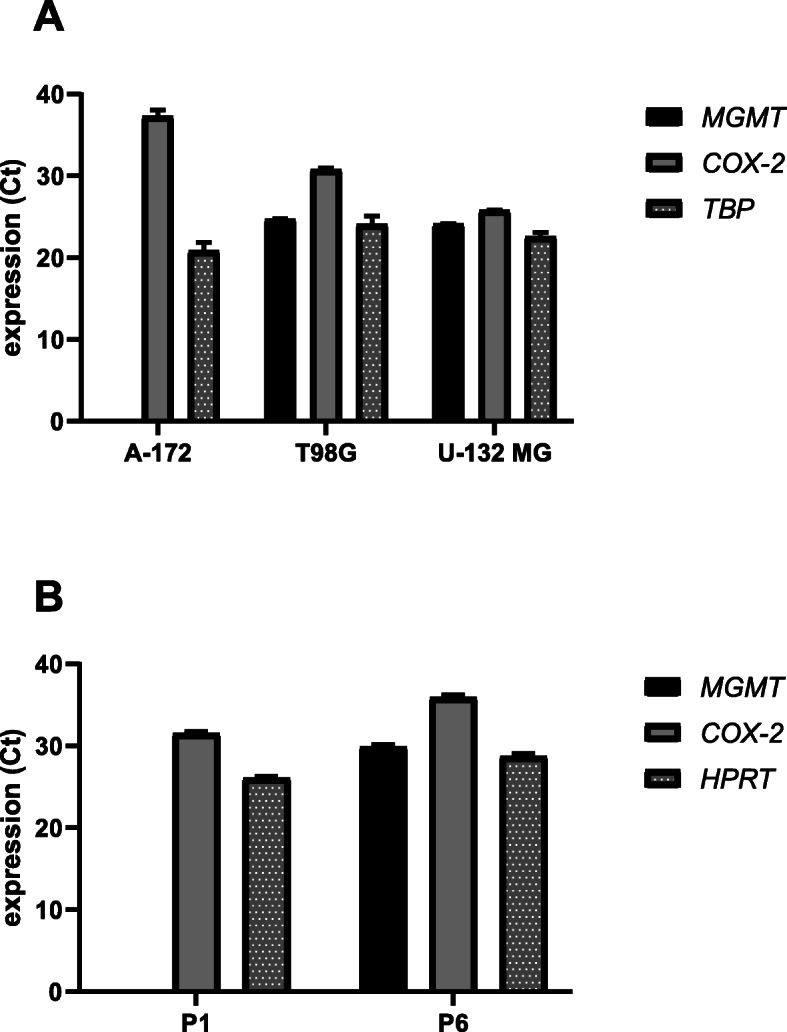
The basic expression levels of *MGMT* and *COX-2* in A-172, T98G, and U-138 MG cell lines (panel **a**), and P1, and P6 cell lines (panel **b**) under normal conditions compared to a house keeping gene (*TBP* and *HPRT*, respectively)

In this study, three commercially available GBM cell lines, A-172 (ATCC-CRL-1620), T98G (92090213) and U-138 MG (ATCC-HTB-16) and two patient-derived primary GBM cell lines, P1 and P6 were used. A-172 and U-138 MG cell lines were purchased from American Type Culture Collection (ATCC), whereas T98G cell line was obtained from the European Collection of Authenticated Cell Cultures (ECACC). The basic expression levels of *MGMT* and *COX-2* in A-172, T98G, and U-138 MG cell lines under normal conditions compared to a housekeeping gene (*TBP*) are presented in Fig. 1a. A-172 cell line is characterized by *MGMT* promoter methylation with no MGMT expression. It is radiosensitive and TMZ sensitive [21, 22]. T98G cell line represents cells with both methylated and unmethylated status of *MGMT* promoter, expressing high MGMT protein level. This cell line is TMZ resistant [21] and radioresistant. T98G cell line also has greater expression of COX-2 than does the radiosensitive cell line, A-172 [22]. Eventually, U-138 MG cell line has unmethylated *MGMT* promoter and high *MGMT* expression. COX-2 is also expressed in this cell line. It is also regarded as TMZ resistant [23].

P1 patient-derived cell line was obtained from 64-year-old female, whereas P6 from 68-year-old male patient. Tissue samples were provided by Chair and Department of Neurosurgery and Neurotraumatology of Poznan University of Medical Sciences based on the ethical approval (KB 534/180). Cells were authenticated by a standard histopathological and molecular analysis done routinely at the Department of Neurosurgery and Neurotraumatology, Heliodor Swiecicki Clinical Hospital, Poznań, Poland. Cells were derived from tumor mass of GBM patients by dissociation using Brain Tissue Dissociation Kit (Miltenyi Biotec, Germany). Our qPCR analysis revealed that *MGMT* is expressed only in P6 cell line, while COX-2 in both of the cell lines. The basic expression levels of *MGMT* and *COX-2* in P1 and P6 cell lines under normal conditions compared to a housekeeping gene (*HPRT*) are presented in Fig. 1b.

A-172, P1, and P6 cells were grown in ATCC-formulated Dulbecco’s modified Eagle’s medium (DMEM) (Merck, Germany), whereas T98G and U-138 MG cell lines were grown in ATCC­-formulated Eagle’s Minimum Essential Medium (EMEM) (Merck, Germany), respectively. These media were supplemented with FBS (Biowest, France) to a final concentration of 10%, as well as antibiotics (penicillin and streptomycin) (Merck, Germany) to the final concentrations of 1%. For the experiments, the amount of FBS was reduced to 5%. Regarding T98G cell line, the medium was also supplemented with 2 mM glutamine, 1% non-essential amino acids, and 1% sodium pyruvate (all purchased from Merck, Germany). Cells were incubated at 37 °C in an atmosphere consisting of 95% air and 5% CO_2_ in a humidified incubator (Memmert, Germany). The passage number was kept low (maximum number 25). Cell morphology and proliferation rate was constantly monitored during the experimental part of the project and never showed any abnormalities. Standard safety procedures were used to avoid cell line contamination with other cell lines and microbes. The LookOut® Mycoplasma PCR Detection Kit (Merck, Germany) was used every two weeks to verify if the cells were mycoplasma free.

### Compounds evaluated

Four COXIBs were used in this experiment, namely: celecoxib, etori-, rofe- and valdecoxib as well as dimethyl derivative of celecoxib – 2,5-DMC. Their chemical structures and IC_50_ values are presented in Table 1. PKF118–310, which blocks the interaction between β-catenin and TCF/LEF transcription factors, was used as a positive control for Wnt/β-catenin inhibition. In contrast, topotecan, a topoisomerase inhibitor, was applied as a positive control in the experiments analyzing apoptosis and cell cycle distribution. To perform various assays, stock solutions in dimethyl sulfoxide (DMSO) were prepared. All the above mentioned compounds, as well as TMZ were purchased from Merck (Germany). For the experiments, all the compounds were diluted ex tempore to the final selected concentration with a complete cell culture medium containing 5% FBS and antibiotics (in the case of T98G cell line also other supplements).

**Table 1:** The chemical structure of the analyzed compounds and their IC_50_ values. The IC_50_ are expressed as mean ± SEM obtained from three independent experiments and calculated based on the dose-response curves assessed by the MTT assay (COXIBs and 2,5-DMC), or taken from the literature (TMZ)

Compound	IC50 [μM] ± SEM			Chemical structure
	A-172	T98G	U-138 MG	
Celecoxib	41 ± 7.11	49 ± 1.34	74 ± 0.99	
2,5-dimethylcelecoxib (2,5-DMC)	23 ± 2.25	32 ± 4.86	33 ± 1.72	
Etoricoxib	> 100	> 100	> 100	
Rofecoxib	> 100	> 100	> 100	
Valdecoxib	> 100	> 100	> 100	
Temozolomide (TMZ)	< 100	> 1000	387.3	

### MTT test

The effect of selected COXIBs, 2,5-DMC and PKF118–310 on the viability of A-172, T98G and U-138 MG cells was assessed by the MTT assay. Briefly, 10,000 cells/well were seeded on 96-well plates and incubated for 24 h. Afterward, tested compounds in various concentrations were added; the controls with DMSO were also included. The test was performed for 48 h, and afterward, the cells were exposed for 3 h to MTT solution dissolved in 10% FBS medium (0.5 mg/ml). After the above-mentioned incubation time, MTT solution was removed, and formazan crystals were dissolved in acidic isopropanol. The absorbance was measured at λ = 570 nm and λ = 690 nm on Tecan Infinite M200 microplate reader (Austria). All the experiments were repeated at least three times with four measurements per assay. Based on MTT assay, the concentrations that allow survival of more than 70% of cells were chosen for further analysis.

### Isolation of DNA and RNA, cDNA synthesis & qPCR

Genomic DNA and total RNA were isolated using GeneMATRIX UNIVERSAL DNA/RNA/protein kit (EURx Ltd., Poland) after 48 h of treatment. The isolations were repeated three times. For cDNA synthesis, RevertAid First Strand cDNA Synthesis Kit (ThermoFisher Scientific, USA) was used, according to the protocol provided by the manufacturer. Next, qPCR for genes of interest was performed using Hot FIREPol EvaGreen qPCR Mix Plus (Solis Biodyne, Estonia) and Chromo4 thermal cycler (Bio-Rad Laboratories, USA). The oligonucleotides were synthesized at the Institute of Biochemistry and Biophysics, Polish Academy of Sciences, Warsaw, and their sequences were published before [24, 25]. Primer sequences for *NEDD9* were the following: *forward* 5′-GAACAAGAGGTATATCAGGTG-3′, and *reverse* 5′-TTGAGTGGTATGAGAAGGAG-3′. The mean expression of *TBP* (*TATA-box-binding protein*) and *PBGD* (*porphobilinogen deaminase*) in the analysis of A-172, T98G, and U-138 MG cell lines transcripts and *HPRT1* (*hypoxanthine phosphoribosyltransferase 1*) in the analysis of P1 and P6 cell lines transcripts were used for data normalization. Primer sequences for *HPRT* qPCR analysis were as follows: *forward* 5′-CCTGGCGTCGTGATTAGTGAT-3′, and *reverse* 5′- AGACGTTCAGTCCTGTCCATAA-3′. The ΔΔCt method was used for fold-change calculation. All samples were analyzed in triplicate, and the reaction was repeated twice.

### Analysis of apoptosis

Muse® Caspase-3/7 Assay Kit (Merck, Germany) was used in order to verify if the analyzed compounds are able to induce apoptosis in GBM cells. This fluorescent-based assay relies on the quantitative measurements of apoptotic status based on caspase-3/7 activation analyzed simultaneously with cellular plasma membrane permeabilization and cell death.

Briefly, cells from all three cell lines (100,000 cells of A-172 and T98G cell lines, and 150,000 cells of U-138 MG cell line) were seeded on 6-well plates and incubated for 24 h. Afterward, the analyzed compounds were added in concentrations based on MTT results, and the cells were further incubated for 48 h. 0.5% or 1% DMSO and 100 nM topotecan were used as negative and positive controls, respectively. The subsequent analysis was performed on Muse™ Cell Analyzer according to the manufacturer’s recommendations (Merck, Germany).

### Analysis of cell cycle distribution

The Muse™ Cell Cycle Kit and Muse™ Cell Analyzer (Merck, Germany) was used in order to check the number of cells in each phase of the cell cycle. The assay utilizes propidium iodide-based staining of DNA content to discriminate and measure the percentage of cells in each cell cycle phase (G0/G1, S, and G2/M). The analysis was performed after 48 h incubation with the analyzed compounds, according to the protocol provided by the manufacturer.

### Methylation-sensitive high resolution melting (MS-HRM)

The methylation level of *MGMT* promoter was determined by MS-HRM on Light Cycler®96 (Roche Diagnostics GmbH, Germany) in T98G cell line. First, 500 ng of genomic DNA was bisulfite converted using EZ DNA Methylation Kit (Zymo Research, USA). As positive/methylated and negative/unmethylated controls, we used CpG Methylated HeLa Genomic DNA (New England Biolabs, USA) and CpGenome Universal Unmethylated DNA Set (Merck, Germany), respectively. Standardized solutions with a given DNA methylation percentage (100, 75, 50, 25, 15, 10, 0% of methylated controls in a background of unmethylated DNA) were prepared. MS-HRM was performed using 5 X Hot FIREPol EvaGreen HRM Mix (Solis BioDyne Co., Estonia) under default cycling conditions and with primers previously published by Wojdacz & Dobrovic [26]. After amplification, the HRM profiles were analyzed using Light Cycler®96 Application Software (Roche Diagnostics GmbH, Germany). Samples were run in duplicate in each experiment, and the experiment was repeated twice.

### Western blot analysis

Cells were lysed with RIPA buffer containing protease inhibitory cocktail. Protein concentration was measured using Spectrophotometer (DeNovix, USA), and 50 μg of denaturized protein samples were separated on SDS-PAGE. Proteins were then transferred to activated with methanol polyvinylidene difluoride (PVDF) membrane using wet tank blotting. Unspecific binding was reduced by 5% w/v non-fat dry milk in PBS-T and hCOX2- (SantaCruz Biotechnology, USA), hPGE2 EP4- (SantaCruz Biotechnology), specific antibody incubations were carried out overnight at 4 °C. As loading control hGAPDH- (SantaCruz Biotechnology, USA) specific antibody conjugated to HRP was employed and was incubated for 1 h at RT. Secondary antibodies were directed against the host of primary antibodies and incubated for 2 h at RT. Membranes were developed using SuperSignal™ West Pico PLUS Chemiluminescent Substrate (ThermoScientific, USA), and signals were read on G-box (Syngene, UK). Densitometric data generated for Western blots were obtained using GelPro Analyzer 3.1.

### Statistical analysis

Student’s t test (GraphPad Prism,Version 7.0 for Windows, USA) was used for analysis of the significance of differences between experimental groups and their respective controls, with *p* < 0.05 considered as significant.

## Results

### COXIBs, and particularly 2,5-DMC, decrease the viability of GBM cell lines in a dose-dependent manner

**Fig. 2:**
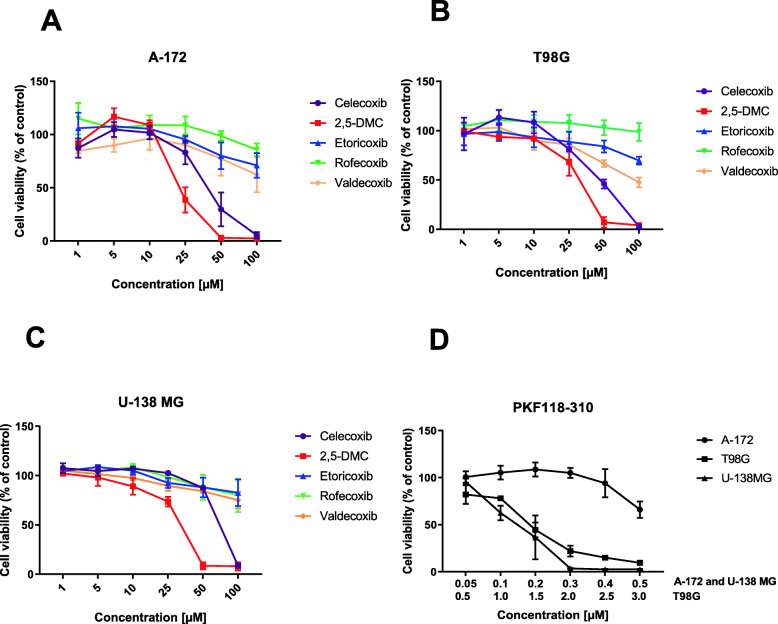
The effect of 48 h treatment with COXIBs on A-172 (panel **a**), T98G (panel **b**) and U-138 MG (panel **c**) cell lines. Panel **d** - the cytotoxicity of PKF118–310. Data presented as mean values ± SEM from three independent experiments

The cell viability analysis with MTT method revealed that sensitivity to COXIBs and 2,5-DMC, tested in a concentrations range of 1–100 μM, does not depend on the status of *MGMT* promoter methylation and does not correlate with TMZ resistance. The highest cytotoxicity was observed after the treatment with 2,5-DMC, followed by celecoxib (Fig. 2a, b and c). Other COXIBs showed a moderate and rather uniform impact on cell viability.

PKF118–310 was cytotoxic to all three cell lines in much lower concentrations – the tested range was 0.05–0.5 μM in A-172 and U-138 MG cell lines, and 0.5–3 μM in T98G cell line (Fig. 2d). U-138 MG cell line was the most sensitive to this compound – 0.3 μM PKF118–310 led to total cell death of this cell line.

In the subsequent experiments, non-toxic concentrations of compounds (allowing the viability level of above 70%) were used.

### COXIBs and 2,5-DMC downregulate β-catenin target genes expression in A-172, T98G and U-138 MG cell lines

**Fig. 3:**
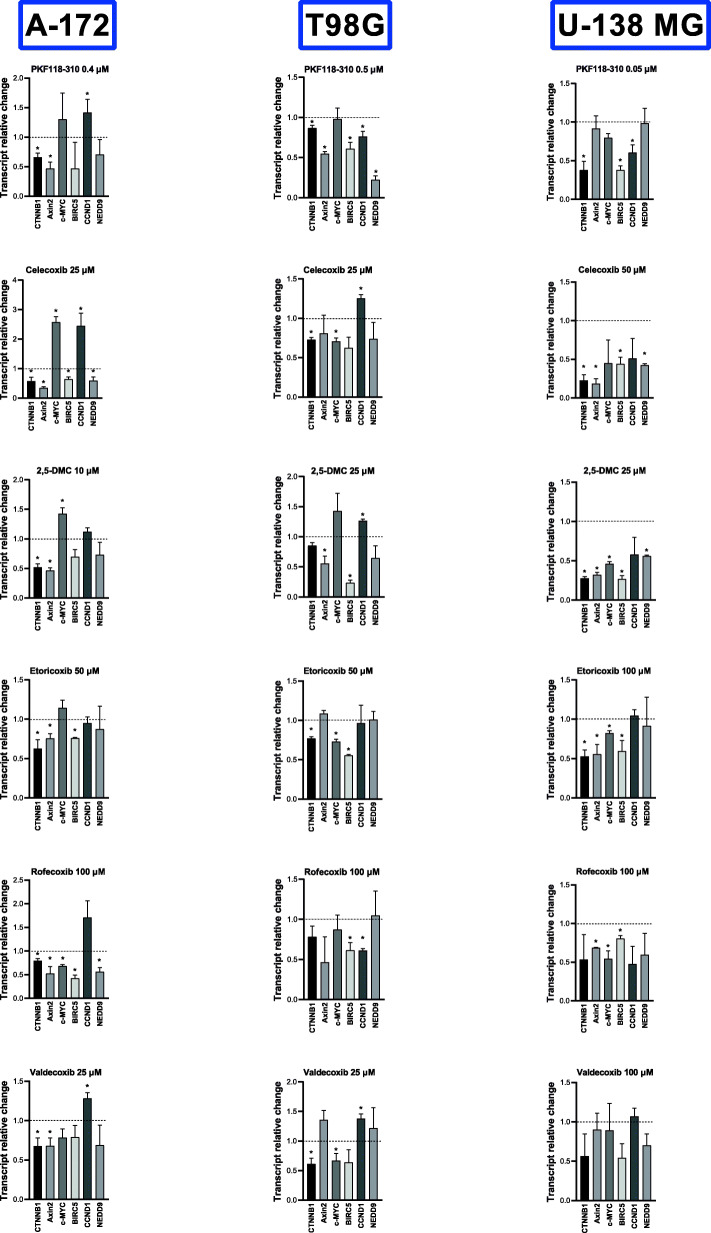
The effect of PKF118–310, COXIBs, and 2,5-DMC on the expression of β-catenin (*CTNNB1*) and its target genes after 48 h of treatment of A-172, T98G, and U-138 MG cell lines. The concentration of compounds used in this assay provided at least 70% of cell viability. Means ± SEM from two separate experiments with three replicates in each are presented. The values were calculated as mRNA level in comparison with control cells treated with DMSO (expression equals 1). The asterisk (*) above the bar denotes a statistically significant difference from the control group, *p* < 0.05

The mRNA level of β-catenin and its target genes, *Axin-2, c-MYC, BIRC5, CCND1,* and *NEDD9* after 48 h of treatment with the analyzed compounds are presented in Fig. 3. The inhibitor of the interaction between β-catenin and TCF/LEF transcription factors - PKF118–310, was used as a positive control. The results illustrate a general trend towards downregulation of both β-catenin transcript level, as well as its target genes level. Nevertheless, A-172, T98G, and U-138 MG cells responded slightly differently to the treatment.

As far as A-172 cell line is concerned, *CTNNB1* encoding β-catenin was downregulated by all the analyzed compounds. PKF118–310 additionally reduced the mRNA level of *Axin2* and surprisingly upregulated *CCND1*. The most effective Wnt pathway inhibitor was however rofecoxib, downregulating not only *CTNNB1*, but also of its target genes: *Axin2*, *c-MYC*, *BIRC5* and *NEDD9*. The second most effective Wnt pathway inhibitor was celecoxib. Still it has to be mentioned that also undesirable up-regulation of *c-MYC* and *CCND1* was observed after the treatment with this compound. 2,5-DMC was also exerting a similar effect concerning *c-MYC*. Etori- and valdecoxib showed moderate effects – downregulated both *CTNNB1* and *Axin2*, as well as *BIRC5* (etoricoxib), but up-regulated *CCND1* (valdecoxib).

Regarding T98G cell line, PKF118–310 Wnt pathway inhibitory effect was the most profoundly seen. All of the analyzed genes (excluding *c-MYC*) were downregulated after the treatment with this compound. It has to be however mentioned that in this experiment, PKF118–310 was used in the highest concentration (0.5 μM) as compared to two other cell lines. Next, the most significant effects were observed after the treatment with etoricoxib, which downregulated *CTNNB1* and two of its target genes, *c-MYC* and *BIRC5*. Celecoxib and its methylated analog showed similar effects as described in A-172 cell line - we observed both desired effects (e.g. downregulation of *BIRC5* after the treatment with 2,5-DMC), but also potentially dangerous up-regulation of *CCND1* oncogene (after the treatment with celecoxib). Rofecoxib was able to downregulate *CCND1* and *BIRC5*, whereas valdecoxib *CTNNB1* and *c-MYC*.

Regarding U-138 MG cell line, PKF118–310 downregulated β-catenin and its two target genes: *CCND1* and *BIRC5*. Among COXIBs, the most profound effects were exerted by 2,5-DMC – this compound downregulated five genes, namely *CTNNB1*, *Axin2*, *c-MYC*, *BIRC5* and *NEDD9*. Celecoxib was the second most effective Wnt pathway inhibitor, reducing the mRNA level of not only β-catenin but also three other of its target genes. Both etori- and rofecoxib downregulated *Axin2* and *c-MYC* (etoricoxib also *CTNNB1*; rofecoxib also *BIRC5*), whereas valdecoxib was not influencing the Wnt pathway target genes expression in this cell line.

### Apoptosis is induced, and cell cycle distribution is altered after the treatment with COXIBs and 2,5-DMC in T98G cell line

**Fig. 4:**
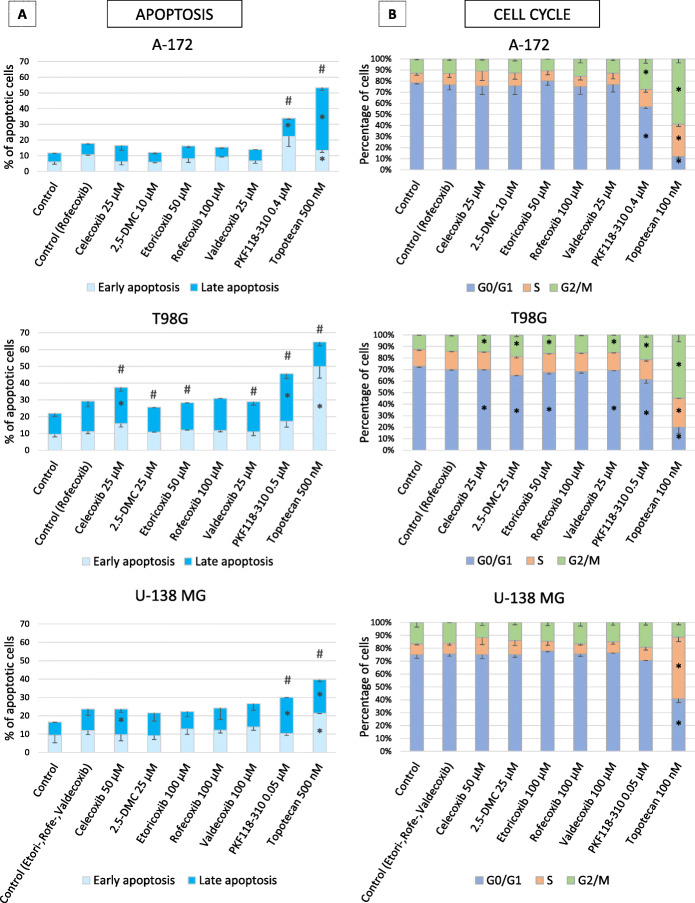
The effect of COXIBs, 2,5-DMC, PKF118–310 and topotecan on apoptosis (panel **a**) and cell cycle distribution (panel **b**) in A-172, T98G and U-138 MG cell lines. The concentration of compounds used in this assay provided at least 70% of cell viability. Mean values ± SD from two independent experiments are shown. In panel **a**, asterisk inside and hashes above bars indicate statistically significant changes for either early or late and total apoptosis, respectively, *p* < 0.05. In panel **b**, asterisk inside bars indicate statistically significant changes, p < 0.05

Wnt/β-catenin pathway and apoptosis are interconnected; thus our next goal was to analyze if COXIBs/2,5-DMC induce apoptosis in GBM cell lines. Figure 4a presents the results of apoptosis analysis, verified based on caspase 3/7 activation and cell membrane permeabilization. 500 nM topotecan, used as the positive control, increased the number of apoptotic cells. PKF118–310 exerted similar effects. COXIBs also induced apoptosis, but only in T98G cells. Celecoxib was, in this case, the most effective, increasing the number of late apoptotic death cells as well as the total number of apoptotic cells. Moreover, 2,5-DMC, etori- and valdecoxib treatment also triggered apoptotic cell death.

The results of the cell cycle distribution analysis illustrate that again only T98G cell line is susceptible to COXIBs treatment (Fig. 4b). In this regard celecoxib, 2,5-DMC, etori- and valdecoxib decreased the number of cells in the G0/G1 phase of the cell cycle, whereas the number of cells in G2/M phase was increased. A similar, but more pronounced effect was observed after the treatment with PKF118–310 and topotecan. As expected, in a concentration of 100 nM, the latter led to cell cycle arrest in the S and G2/M phase in all analyzed cell lines.

### COXIBs and 2,5-DMC do not alter *MGMT* methylation status in T98G cell line

**Fig. 5:**
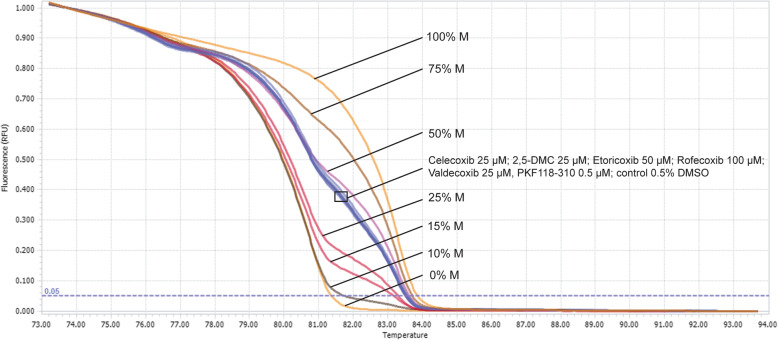
*MGMT* methylation level in DNA isolated from T98G cells determined using MS-HRM analysis after 48 h of treatment with the analyzed COXIBs, 2,5-DMC, PKF118–310 and 0.5% DMSO (control). The concentration of compounds used in this assay provided at least 70% of cell viability. Normalized melting curves of standardized solutions with a given DNA methylation percentage (indicated with ‘M’) in a background of unmethylated DNA are presented together with the results of the analysis. For clarity, one replicate of each analyzed sample/control is shown

*MGMT* methylation is an important determinant of GBM patients response to TMZ [27]. Thus, the impact of COXIBs on *MGMT* methylation level was analyzed in T98G cell line. This cell line was chosen for this experiment since the above-mentioned tests showed the highest sensitivity to the COXIBs treatment compared to the other two cell lines. Figure 5 shows that after 48 h treatment with COXIBs, 2,5-DMC or PKF118–310 *MGMT* methylation level is not altered, as compared to the results for 0.5% DMSO (control). In all cases, the resulting methylation level was close to 50% methylation.

### COXIBs and 2,5-DMC downregulate β-catenin target genes expression in patient-derived primary GBM cell lines P1 and P6

**Fig. 6:**
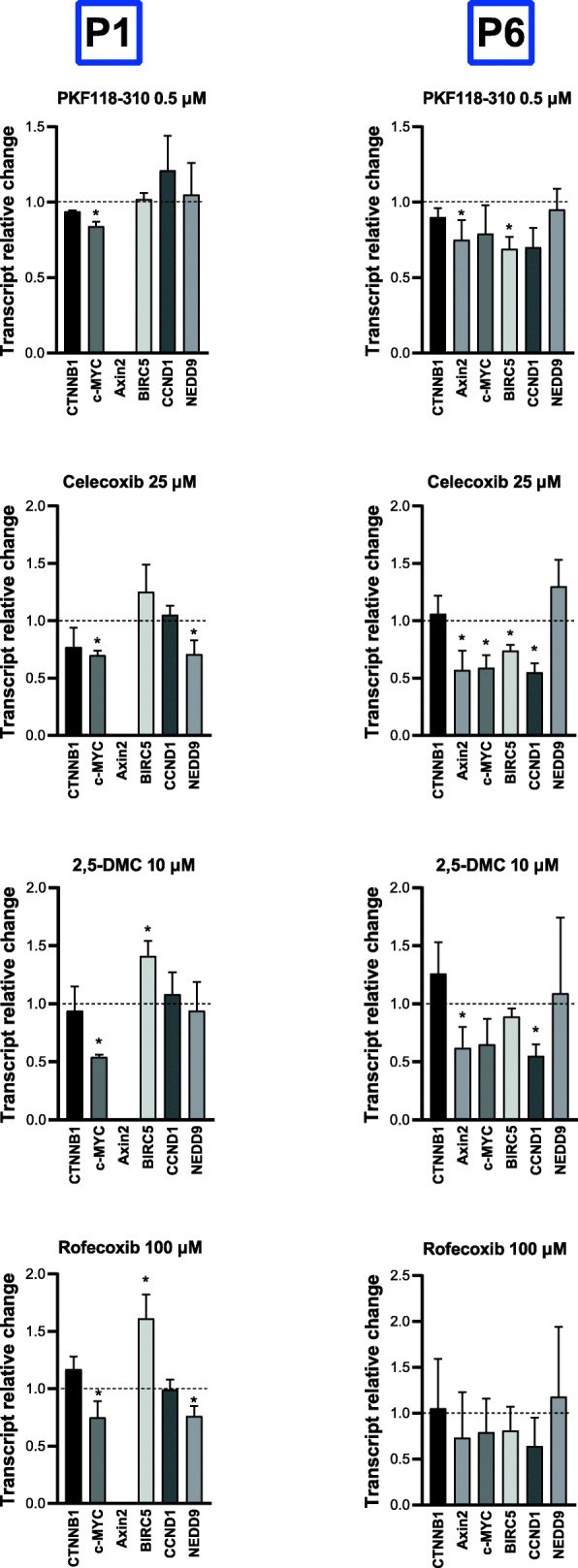
The effect of PKF118–310, celecoxib, 2,5-DMC and rofecoxib on the expression of β-catenin (*CTNNB1*) and its target genes after 48 h of treatment of P1 and P6 patient-derived primary cell lines. The concentration of compounds used in this assay provided at least 70% of cell viability. Means ± SEM from two separate experiments with three replicates in each are presented. The values were calculated as mRNA level in comparison with control cells treated with DMSO (expression equals 1). The asterisk (*) above the bar denotes statistically significant difference from the control group, p < 0.05

Next, based on the qPCR analysis of β-catenin target genes expression using commercial GBM cell lines, we chose the three most effective compounds - celecoxib, 2,5-DMC and rofecoxib, and verified their impact on two patient-derived primary GBM cell lines – P1 and P6 after 48 h of treatment (Fig. 6). Our results show that even though the expression of β-catenin was neither downregulated by PKF118–310, nor by COXIBs or 2,5-DMC, the mRNA levels of β-catenin target genes were often diminished. In this regard, celecoxib downregulated *c-MYC* and *NEDD9* in P1 cell line, and also *Axin2*, *c-MYC, CCND1,* and *BIRC5* in P6 cell line. Its dimethyl derivative, 2,5-DMC was slightly less effective – in P1 cell line it downregulated *c-MYC* expression, but also upregulated *BIRC5*. Moreover, the mRNA levels of *Axin2* and *CCND1* were reduced after the treatment with this compound in P6 cell line. On the other hand, rofecoxib was effective only in P1 cell line – it reduced the expression of *c-MYC* and *NEDD9*, but increased *BIRC5*.

### COXIBs and 2,5-DMC downregulate the expression of COX-2 and PGE2 EP4 receptor

**Fig. 7:**
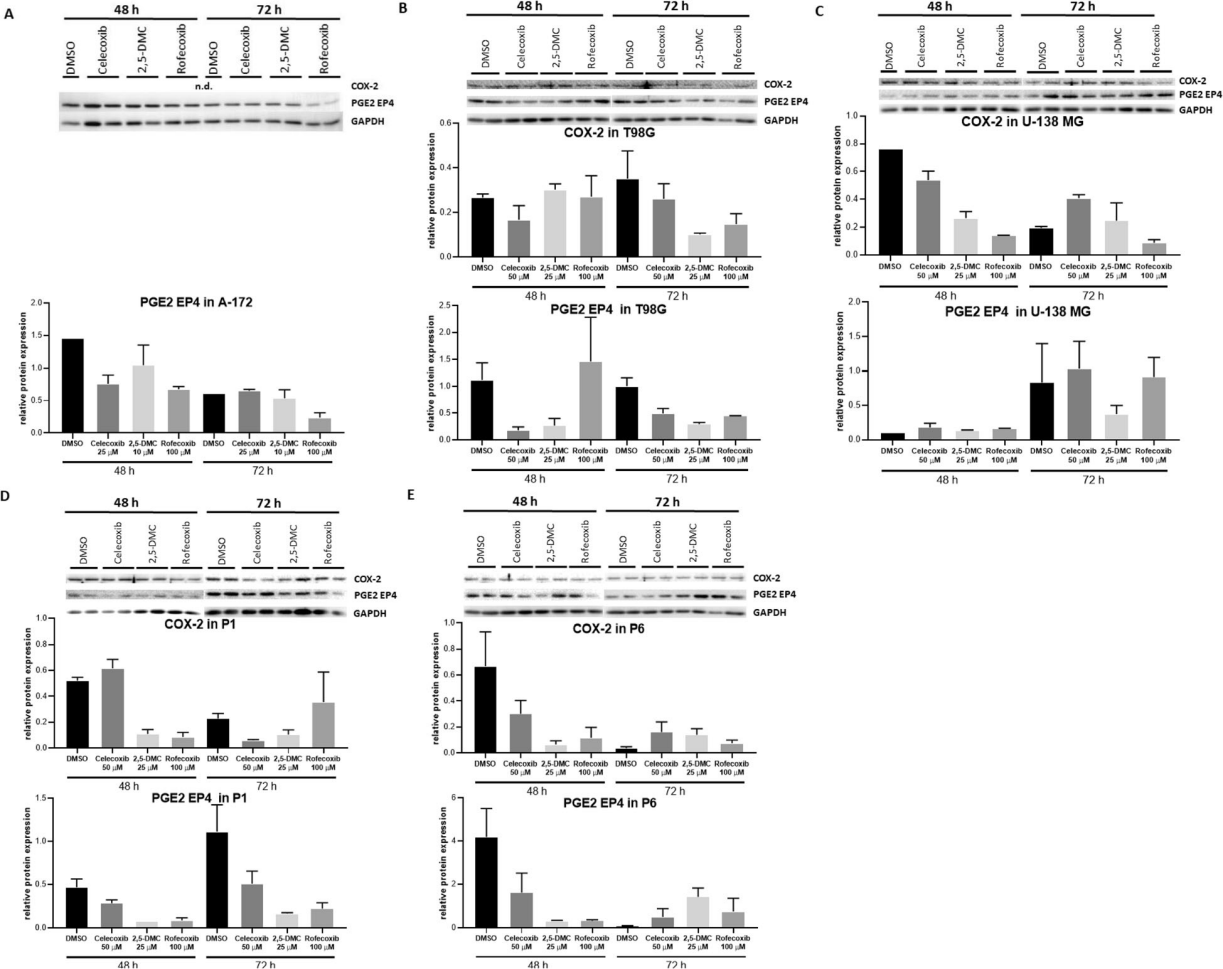
Analysis of COX-2 and PGE2 EP4 expression in different cell lines. A-172 (**a**), T98G (**b**), U-138 MG (**c**), P1 (**d**) and P6 (**e**) cells were treated with celecoxib, 2,5-DMC and rofecoxib for 48 and 72 h. The concentration of compounds used in this assay provided at least 70% of cell viability. PGE EP4 and COX-2 protein levels were evaluated by Western blot. DMSO was used as a control. GAPDH was used as loading control. Graphs represent densitometric analysis of protein of interest to loading control

In addition to COX-2 inhibition, COX-2-independent mechanisms contribute to the anti-tumorigenic effects of COXIBs. To verify if the impact on Wnt/β-catenin pathway, apoptosis, and cell cycle are caused more by COX-2 dependent, or COX-2 independent mechanisms, we analyzed the basal expression of COX-2 in A-172, T98G, U-138 MG, P1 and P6 cell lines. Based on qPCR results, our study shows that *COX-2* was not expressed in A-172 cell line while its expression in T98G, U-138 MG, P1 and P6 cell lines was relatively weak. Western blot analysis confirmed these findings. Next, we checked if the protein level of COX-2 changes during the course of treatment with celecoxib, 2,5-DMC and rofecoxib. The results showed that the impact of celecoxib, 2,5-DMC, and rofecoxib was cell line dependent and changed over time; thus a slight downregulation of COX-2 could be observed in T98G, U-138 MG, P1 after rofecoxib and in P1 after celecoxib treatment (Fig. 7). COX-2 protein level was diminished after the treatment with celecoxib, 2,5-DMC and rofecoxib in T98G cell line (72 h after treatment), and U-138 MG (48 h after treatment). In respect of P1 cells, mild decrease of COX-2 level was observed after the treatment with 2,5-DMC in both analyzed time points and celecoxib after 72 h.

Since PGE2 EP4 receptor is critical in mediating COX-2/PGE2 responses, we wanted to evaluate if its expression is altered after the treatment with celecoxib, 2,5-DMC, and rofecoxib. The results show a general trend towards downregulation of PGE2 EP4 after the treatment with celecoxib, 2,5-DMC and rofecoxib, especially in T98G and P1 cell lines (Fig. 7). Moreover, similar results were observed in P6 cell line. No impact on PGE2 EP4 protein level was observed in A-172 (with the exception for rofecoxib, which downregulated protein expression 72 h after treatment). U-138 MG cell line in both analyzed time points was not responsive.

Full-length blots are presented in Supplementary Fig. 1.

### Combined TMZ and COXIBs/2,5-DMC treatment also attenuate Wnt/β-catenin pathway

**Fig. 8:**
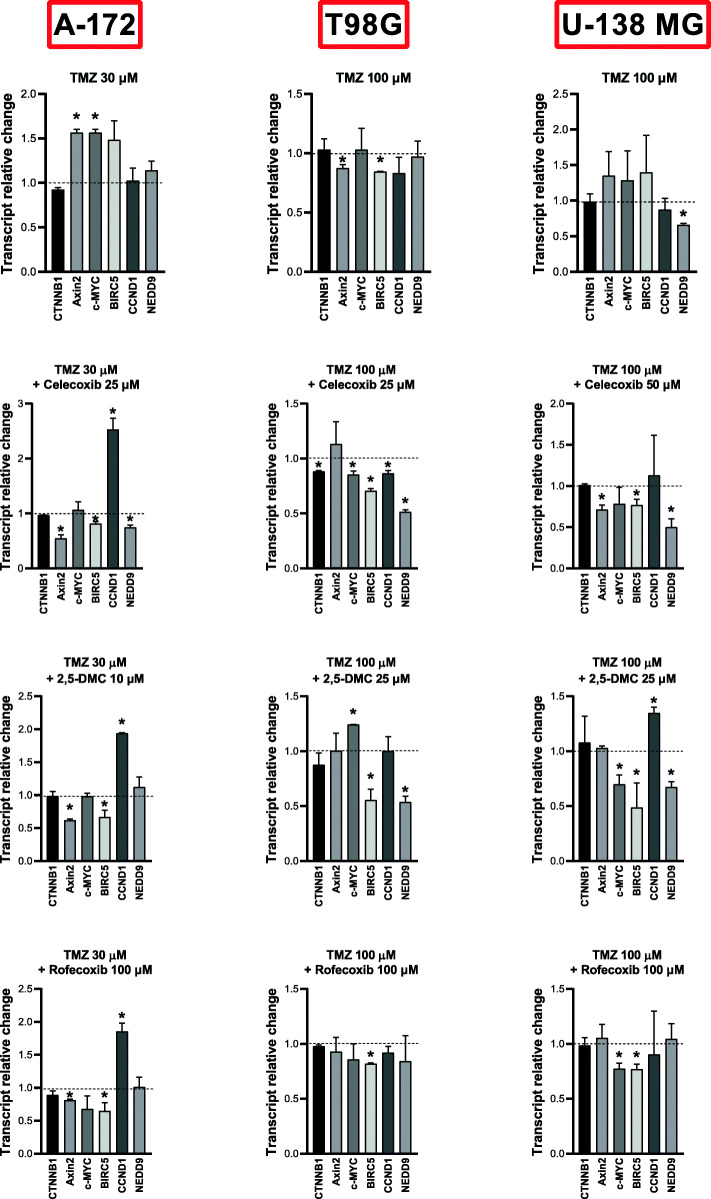
The effect of combined TMZ and celecoxib, or 2,5-DMC, or rofecoxib on the expression of β-catenin (*CTNNB1*) and its target genes after 48 h of treatment of A-172, T98G and U-138 MG cell lines. The concentration of compounds used in this assay provided at least 70% of cell viability. Means ± SEM from two separate experiments with three replicates in each are presented. The values were calculated as mRNA level in comparison with control cells treated with DMSO (expression equals 1). The asterisk (*) above the bar denotes statistically significant difference from the control group, p < 0.05

Besides surgical resection and radiotherapy, chemotherapy with TMZ is most often used in GBM treatment [27]. Thus, we wanted to evaluate if combined TMZ and COXIBs/2,5-DMC exert Wnt pathway downregulatory effect in GBM cell lines after 48 h of treatment. Three chosen compounds, celecoxib, 2,5-DMC and rofecoxib were evaluated together with 30 μM TMZ (chemosensitive A-172 cell line), or 100 μM TMZ (chemoresistant T98G and U-138 MG cell lines). As presented in Fig. 8, all three cell lines reacted slightly differently to the treatment, but a general trend towards downregulation of the β-catenin target genes, especially *BIRC5* and *NEDD9*, was maintained.

In regard to A-172 cell line, the combined treatment with TMZ and celecoxib downregulated *Axin2, BIRC5* and *NEDD9.* The combination of TMZ with 2,5-DMC or rofecoxib had similar effects - strong downregulation of *Axin2* and *BIRC5* was observed. However, all three combinations resulted also in upregulation of *CCND1.* This phenomenon was also seen when single compounds were used, so it can be attributed to COXIBs/2,5-DMC, rather than TMZ. Noteworthy, the level of *c-MYC* proto-oncogene, significantly upregulated after TMZ treatment, was lowered to the level of DMSO treated control, in the presence of COXIBs/2,5-DMC.

In T98G cell line, the most beneficial effects were observed after the treatment with TMZ and celecoxib – five out of six β-catenin target genes were downregulated. The combination of TMZ and 2,5-DMC resulted in the reduction of *BIRC5* and *NEDD9* expression, whereas only *BIRC5* level was diminished after the treatment with both TMZ and rofecoxib.

Similar results were obtained for U-138 MG cell line. In this case, TMZ combined with either celecoxib or 2,5-DMC downregulated *BIRC5* and *NEDD9*. Moreover, TMZ and celecoxib had also impact on *Axin2* expression, whereas TMZ and 2,5-DMC on *c-MYC* (in both cases the mRNA level was diminished). However, similarly to A-172 cell line, *CCND1* was upregulated after the treatment with both combinations (in case of celecoxib there was a trend towards upregulation, but in regard to 2,5-DMC the result was statistically significant). On the other hand, the combination of TMZ and rofecoxib downregulated the mRNA levels of two genes: *c-MYC* and *BIRC5*.

**Fig. 9:**
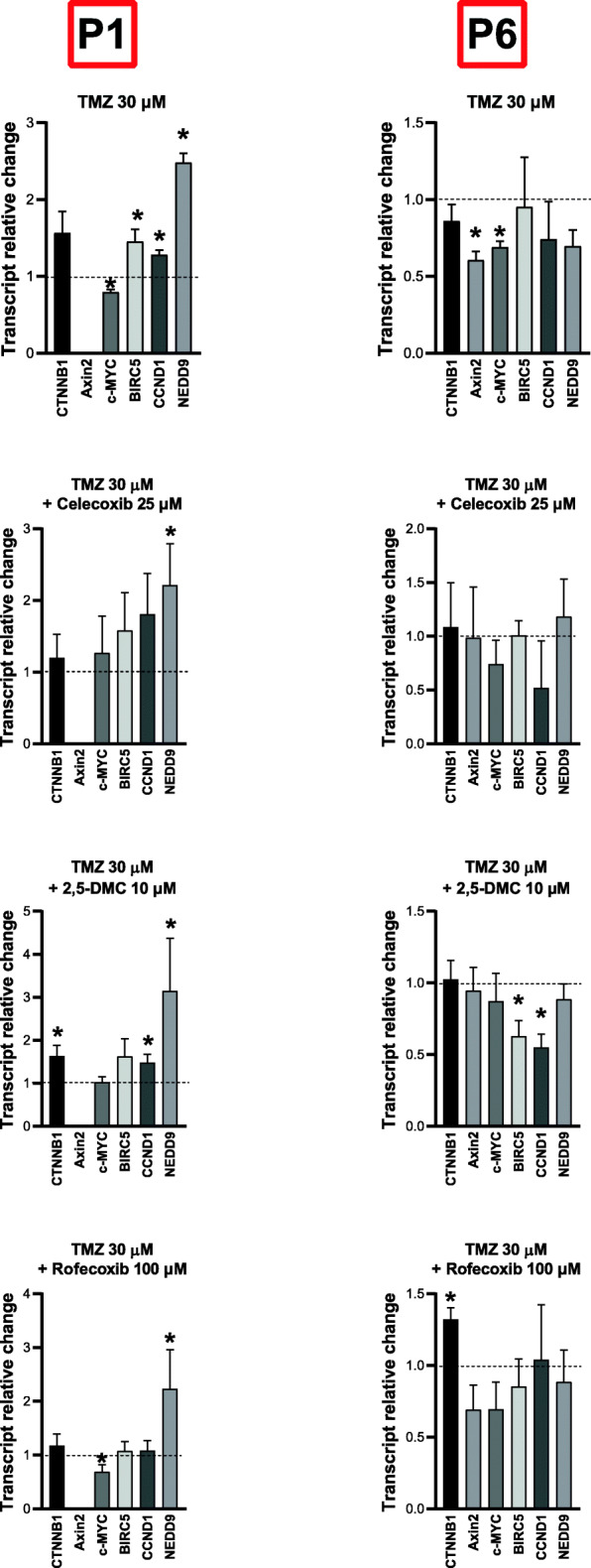
The effect of combined TMZ and celecoxib, or 2,5-DMC, or rofecoxib on the expression of β-catenin (*CTNNB1*) and its target genes after 48 h of treatment of patient-derived primary P1 and P6 cell lines. The concentration of compounds used in this assay provided at least 70% of cell viability. Means ± SEM from two separate experiments with three replicates in each are presented. The values were calculated as mRNA level in comparison with control cells treated with DMSO (expression equals 1). The asterisk (*) above the bar denotes statistically significant difference from the control group, p < 0.05

Next, we wanted to verify if Wnt/β-catenin pathway is inhibited in patient-derived primary P1 and P6 cell lines, when 30 μM TMZ is combined with 25 μM celecoxib, 10 μM 2,5-DMC or 100 μM rofecoxib (Fig. 9). TMZ treatment only in P1 cell line, down-regulated *Axin2*, but upregulated *BIRC5*, *CCND1,* and *NEDD9*. Noteworthy, the latter was up-regulated also when the combinations of TMZ with celecoxib, 2,5-DMC and rofecoxib were evaluated. Furthermore, besides previously mentioned *NEDD9* up-regulation no gene expression changes were observed when TMZ was combined with celecoxib in this cell line. Similar results were observed in P6 cells. As far as TMZ and 2,5-DMC combination is concerned, the beneficial downregulation of *BIRC5* and *CCND1* was observed in P6 cells. The latter gene, as well as *CTNNB1* were however up-regulated in the same experimental setting in P1 cells. TMZ and rofecoxib was also inducing β-catenin up-regulation in P6 cells, but downregulated *Axin2* in P1 cell line.

## Discussion

Recent literature points out that Wnt/β-catenin pathway hyperactivation is one of the most important features of GBM [28–30]. Upregulation of this pathway results in the promotion of cell proliferation, inhibition of apoptosis, and cell invasion [31–33]. Wnt/β-catenin signaling is also responsible for the development of resistance to currently approved therapies and results in poor prognosis [7]. Furthermore, inflammation is crucial for GBM progression. Wnt/β-catenin pathway and COX-2/PGE2/EP4 pathway are therefore regarded as two crucial targets in GBM treatment. A growing amount of data also suggest that the cancer-protective effect of COXIBs is both due to their COX-2 inhibiting potential as well as the ability to interfere with the Wnt/β-catenin pathway, independently of COX-2/PGE2 pathway [28].

**Fig. 10:**
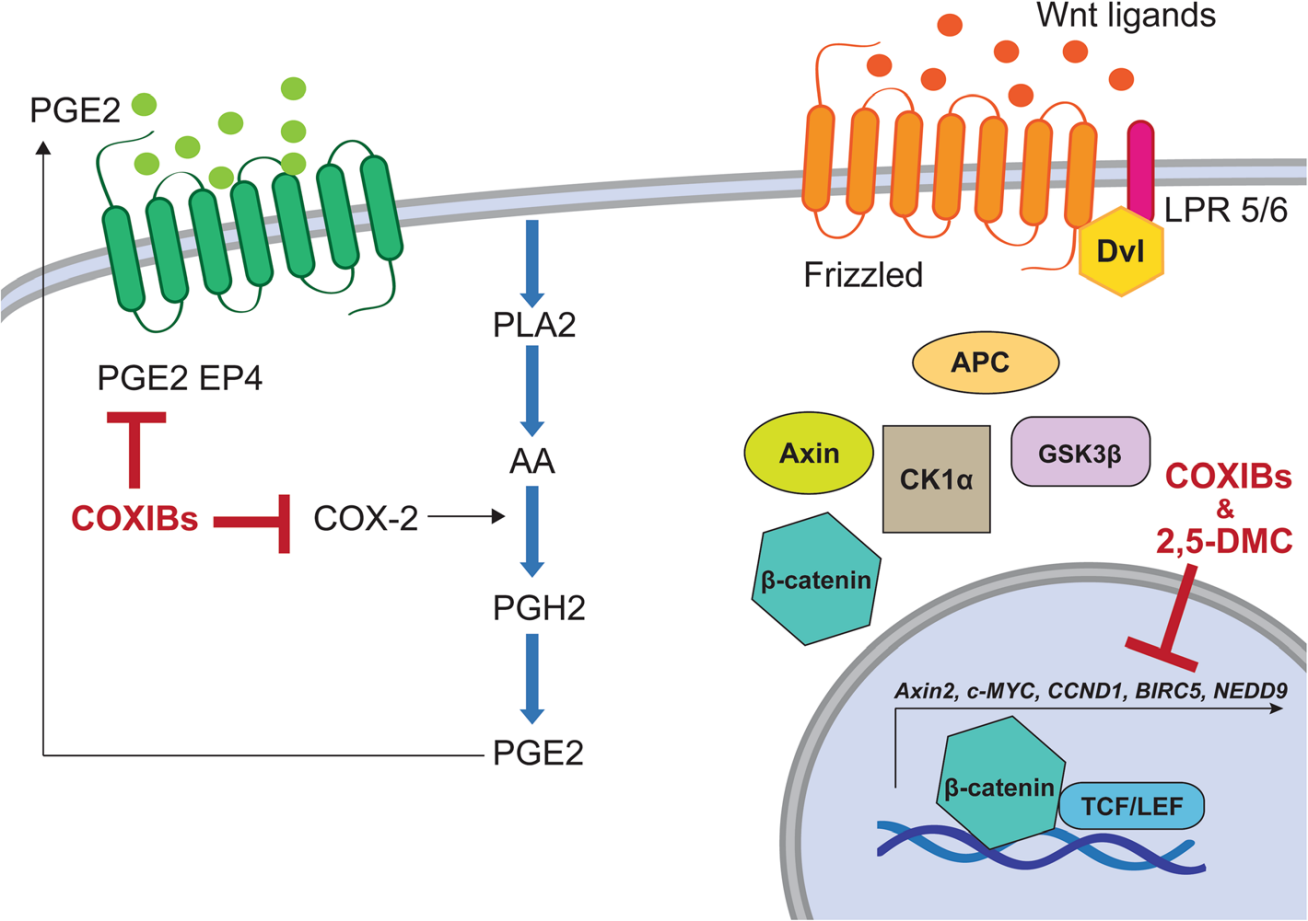
COXIBs and 2,5-DMC counteract the hyperactivated Wnt/β-catenin pathway and COX-2/PGE2/EP4 signaling in glioblastoma cells. Arachidonic acid (AA) is released from cell membrane via the action of phospholipase A2 (PLA2) and converted to prostaglandin H2 (PGH2) through the activity of COX-2 enzyme. PGH2 is further transformed to five types of prostanoids, including PGE2. Cellular responses of PGE2 are exerted via four G-protein coupled receptors, including EP4 receptors. The COX-2/PGE2/EP4 axis plays crucial role in GBM development and progression, but it can be counteracted by COXIBs and 2,5-DMC. As presented in our study, COXIBs and 2,5-DMC downregulate the expression of both COX-2 and PGE2 EP4 (important to note - 2,5-DMC is not indicated in this part the Figure for clarity, as it is does not inhibit the enzymatic activity of COX-2). Moreover, the Wnt/β-catenin pathway is hyperactivated in GBM, mostly due to the epigenetic silencing of its extracellular antagonists. In the presence of Wnt ligand, the Frizzled receptors make a complex with low-density lipoprotein receptor-related protein 5 or 6 (LRP5/6) and Disheveled (Dvl), resulting in inhibition of β-catenin phosphorylation and thus ensuing β-catenin stabilization. The degradation complex, consisting of Axin, GSK3β, CK1α and APC is not being formed. Subsequent translocation of β-catenin to the nucleus activates TCF/LEF-mediated transcription of β-catenin target genes including *Axin2*, *c-MYC*, *CCND1*, *BIRC5*, and *NEDD9*. COXIBs and 2,5-DMC downregulate their expression, attenuating the Wnt pathway

Thus, we focused on the impact of celecoxib, 2,5-DMC, etori-, rofe-, and valdecoxib, on GBM cells. The results of this study show that not only celecoxib, but also 2,5-DMC (lacking COX-2 inhibitory activity) as well as etori-, rofe- and valdecoxib can be regarded as promising compounds with anti-GBM properties. Their mechanism of action, determined based on the results of this study, is shown in Fig. 10. It has to be mentioned that detrimental cardiovascular side effects exerted by rofe- and valdecoxib, which were the reason of their withdrawal from the market, are a serious concern. However, in the case of GBM, local administration of these drugs can be considered, in the way of e.g. biopolymers or nanocarriers loaded to the tumor cavity after resection, minimizing the risk of dangerous side effects observed after systemic delivery.

We found that, in regard to the impact of COXIBs on GBM cell viability, the sensitivity of GBM cell lines to COXIBs does not depend on the *MGMT* promoter methylation status and does not correlate with the TMZ resistance. In this regard, A-172, T98G, and U-138 MG cell lines reacted in a similar way to the treatment with the analyzed compounds. The two most cytotoxic compounds were 2,5-DMC and celecoxib. Similar results were published regarding celecoxib’s treatment of GBM cell lines U87 and U251 – in this case celecoxib reduced not only cell proliferation, but also colony formation, and cell invasion [34]. The fact that celecoxib and 2,5-DMC have the strongest impact on GBM cells can lead to the conclusion that the effect of selected agents is not restricted solely to COX-2 inhibitory pathway, but can result from their ability to stimulate cell death via COX-2 independent mechanisms [35, 36]. That is why our next goal was to establish the role of different COXIBs as Wnt pathway inhibitors. To better reflect GBM heterogeneity, we used three commercially available and two patient-derived GBM cell lines for this purpose.

We found that tested cells showed different sensitivities to Wnt pathway inhibitor PKF118–310 - the most sensitive was U-138 MG. Our study demonstrates that also COXIBs are effective Wnt pathway inhibitors, but their influence is highly dependent on the cell line used. In this regard, rofecoxib was most effectively inhibiting β-catenin and its target gene expression in A-172 cell line, etoricoxib in T98G cell line, whereas 2,5-DMC in U-138 MG cell line. On the other hand, celecoxib was the most efficient, as compared to 2,5-DMC and rofecoxib, when tested on patient-derived primary cell lines.

Furthermore, Wnt/β-catenin signaling pathway regulates apoptosis through many different mechanisms, including Wnt/BMP (bone morphogenic protein) interaction, SFRP2 (secreted Frizzled-related protein-2) expression, and β-catenin [37]. In this study, the effect of selected compounds on apoptosis was determined via their ability to activate caspase 3/7. Thus, it can be expected that agents which downregulate Wnt/β-catenin signaling pathway are more likely to promote apoptosis [37]. Interestingly, the results of this study show that the effect of COXIBs on apoptotic cell death is cell line dependent, and was restricted only to T98G cell line. This finding clearly demonstrates that GBM is highly heterogenous, but also shows promising results that radio- and chemoresistant cells, with high expression of COX-2 respond well to COXIBs/2,5-DMC treatment. Celecoxib, 2,5-DMC, etori- and valdecoxib increased the percentage of total apoptotic cells in the analyzed population. Similar findings were reported by several other groups [35, 38, 39]. The possible mechanism of apoptosis induction by 2,5-DMC was shown in a study by van Roosmalen et al. [40]. 2,5-DMC is an endoplasmic reticulum stress-inducing agent which enhances TRAIL-induced apoptosis in GBM cells. Moreover, concerning celecoxib, several studies confirmed that its pro-apoptotic effect is not related to its COX-2 inhibitory action, but is exerted rather via the antagonistic effect on the anti-apoptotic proteins Mcl-1 and survivin [35, 39]. Our study confirms the above-mentioned hypothesis, since the reduction of *BIRC5*, encoding survivin was observed after the treatment with 2,5-DMC, etori- and rofecoxib. These findings also confirm the importance of the correlation between apoptosis induction and the reduction of survivin levels via Wnt/β-catenin pathway inhibition in GBM cells [35, 39].

Moreover, the effect of selected compounds on apoptosis may be correlated to their impact on cell cycle distribution. A significant similarity between these two phenomena was observed in the case of T98G cell line. The same compounds - celecoxib, 2,5-DMC, etori-, and valdecoxib reduced the number of cells in G0/G1 and increased the fraction of cells in G2/M phases similarly to the effect of a positive control of the assay – topotecan and also PKF118–310. In regard to rofecoxib, the induced changes in cell cycle distribution can be attributed to its effect on *CCND1* gene expression, since cyclin D1, encoded by this gene is necessary for G1/S transition.

It has recently been established that in gastric cancer cells, PGE2 induces DNMT3B expression and activity, which in turn results in a higher level of *MGMT* promoter methylation [15]. However, in our study COXIBs and 2,5-DMC did not alter *MGMT* methylation status after 48 h of treatment. Perhaps a longer incubation time is required to observe this phenomenon. Such an epigenetic change would be beneficial in regard to TMZ treatment. Interestingly, Wickström et al. [19] have shown that β-catenin activates the expression of *MGMT* through direct interaction with TCF/LEF transcription factor binding sites located in the 5′-upstream regulatory region of the *MGMT* gene. Moreover, in this study celecoxib treatment reduced the levels of MGMT in a mouse model [19].

Elevated levels of COX-2 in GBM are highly correlated with many aggressive aspects of the disease, including a high rate of GBM cell proliferation, higher tumor grade, and poor prognosis [13]. Thus, our next goal was to analyze if the treatment with COXIBs/2,5-DMC influences the level of expression of COX-2 protein. Interestingly, our data show that 2,5-DMC, despite its inability to block COX-2 enzymatic activity, is able to reduce its expression, most likely inhibiting also the COX-2/PGE2 signaling. Moreover, our data demonstrate a general trend towards COX-2 downregulation as a response not only to 2,5-DMC, but also rofecoxib, and to a lesser extent also celecoxib treatment. This phenomenon was particularly evident in U-138 MG, P1, and P6 cell lines when the analysis was performed 48 h after treatment, and also in T98G cells, 72 h after treatment. Similar results were recently published by Yang et al. [41], who observed significantly COX-2 downregulation in response to rofecoxib treatment.

Recently, PGE2 has emerged as a principal mediator for COX-2 cascade-driven gliomagenesis [42]. PGE2 mediates its actions via four G-protein coupled receptors – EP1–EP4. A critical role in GBM cell proliferation and resistance to radiation therapy is attributed to EP4 receptor, as its signaling pathways control cell proliferation, invasion, apoptosis, and angiogenesis in GBM cells [43]. Interestingly, our study demonstrates that the protein level of PGE2 EP4 receptor is diminished, especially after the treatment with 2,5-DMC and rofecoxib, and also to a lesser extent, celecoxib. This phenomenon was evident, especially in T98G and P1 cell lines and also in P6 cell line in the first analyzed time-point. Selectively targeting EP4 receptors might provide an alternative therapeutic strategy for GBM, thus this issue requires further investigation.

TMZ is the most common alkylating agent used in GBM treatment [27]. Thus, we wanted to verify if the combination of TMZ with COXIBs/2,5-DMC has an inhibitory effect on Wnt pathway. Our results show that in general, such combinations work similarly, but the particular genes affected differ slightly between the treatments and among different cell lines. Overall, the combination of TMZ and celecoxib was the most effective in the commercial GBM cell lines, but also the TMZ and 2,5-DMC or TMZ and rofecoxib were able to inhibit β-catenin target genes expression.

## Conclusions

To conclude, the results of our study show that COXIBs, as a class of drugs, produce variable, but beneficial effects and can be regarded as good anti-GBM therapeutics. Celecoxib and 2,5-DMC were the most cytotoxic, but other COXIBs also showed good antitumor activities, including Wnt/β-catenin pathway target genes inhibition. Our findings also support the theory that the Wnt/β-catenin pathway is attenuated by COXIBs and 2,5-DMC irrespective of the COX-2 expression profile or *MGMT* methylation status of the treated cells. Moreover, we found that β-catenin target genes were down-regulated by COXIBs and 2,5-DMC also when these drugs were combined with TMZ. In regard to the potential cardiotoxicity of rofe- and valdecoxib, their administration into the tumor niche should be a solution. Novel drug delivery systems could limit the effects on the entire organism, enhancing the effect on the remaining glioma stem cells after the resection of this diffusely growing tumor. In our study, the T98G cell line was the most prone to apoptosis induction and cell cycle arrest, indicating that COXIBs and 2,5-DMC could potentially be valuable drugs for radio- and chemoresistant GBM patients. Our study also demonstrates that the COX2/PGE2/EP4 signaling can be attenuated by COXIBs and 2,5-DMC. Therefore it needs to be emphasized that more pre-clinical and clinical studies addressing combinational COXIBs/2,5-DMC and chemotherapy treatment, as well as targeted brain delivery are required to fully establish the role of these compounds in anti-GBM treatment.

## Supplementary Information

**Additional file 1: Supplementary Fig. 1.** The original images for all relevant western blot analysis of COX-2 and PGE2 EP4 expression in different cell lines. A-172 (A), T98G (B), U-138 MG (C), P1 (D) and P6 (E) cells were treated with celecoxib, 2,5-DMC and rofecoxib for 48 and 72 h. The concentration of compounds used in this assay provided at least 70% of cell viability. PGE2 EP4 and COX-2 protein levels were evaluated by western blot. DMSO was used as a treatment control. GAPDH was used as loading control. The closest protein standard marker band for respected analyzed protein is marked with expected size in kDa. Mostly the protein standard marker is added as separate picture taken. Black boxes represent the cropped bands taken for the analysis and presented in the main body of the manuscript. These were taken into densitometric analyses.

## Data Availability

The datasets used and/or analyzed during the current study are available from the corresponding author on reasonable request.

## Abbreviations

COX-2: Cyclooxygenase 2
COXIBs: The selective COX-2 inhibitors
2,5-DMC: 2,5-dimethylcelecoxib
DNMT3B: DNA methyltransferase 3B
GBM: Glioblastoma multiforme
MGMT: O^6^-methylguanine-DNA methyltransferase
MS-HRM: Methylation-sensitive high resolution melting
PGE2: Prostaglandin E2
PGE2 E4: Prostaglandin E2 receptor, subtype E4
TMZ: Temozolomide
