# Supplementary figures and images for: COXIBs and 2,5-dimethylcelecoxib counteract the hyperactivated Wnt/β-catenin pathway and COX-2/PGE2/EP4 signaling in glioblastoma cells
Source: BMC Cancer. 2021 May 3;21:493. doi: 10.1186/s12885-021-08164-1 (PMC8091781; doi:10.1186/s12885-021-08164-1)

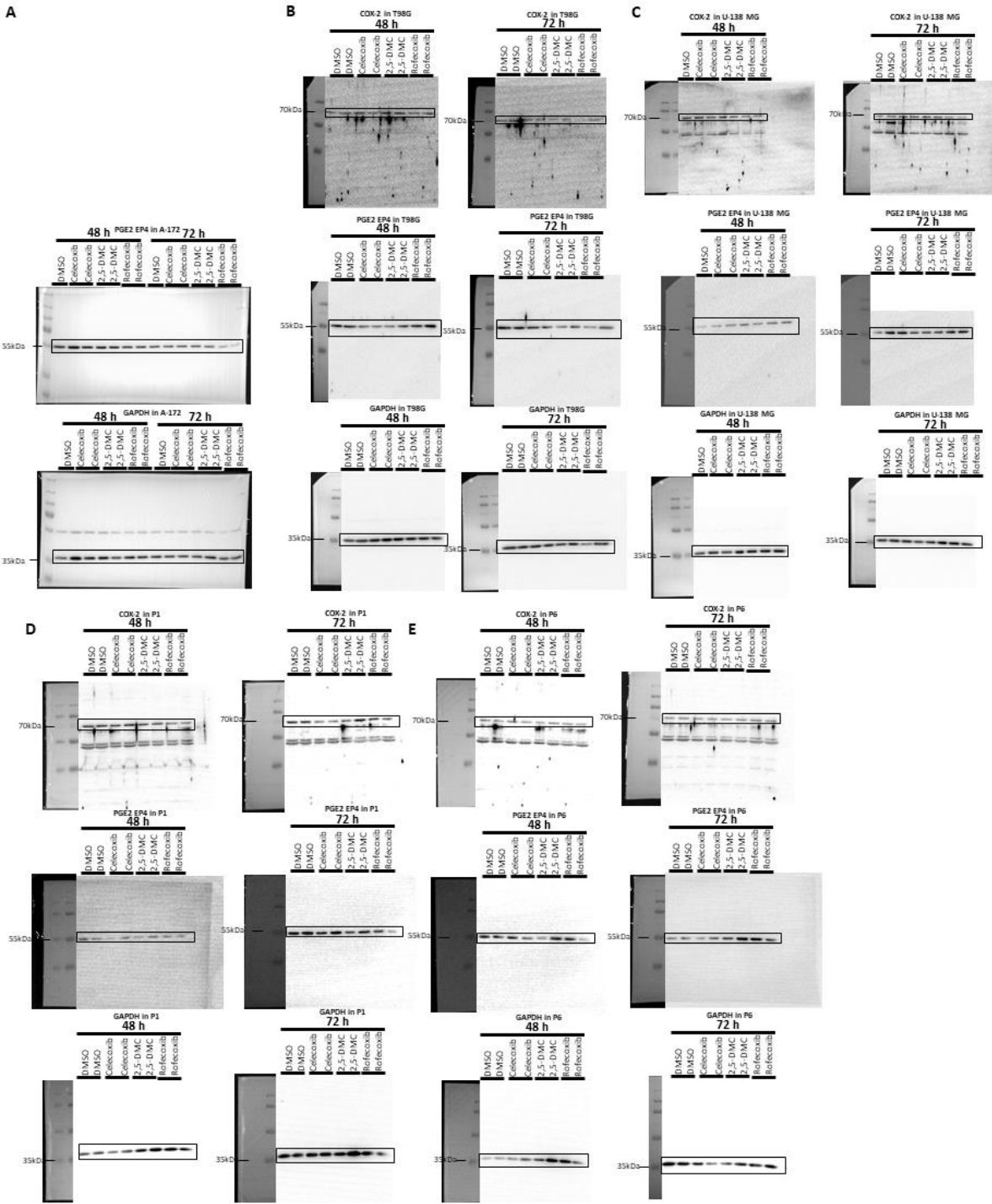

Supplement: Supplementary file 1 — Additional file 1: Supplementary Fig. 1. The original images for all relevant western blot analysis of COX-2 and PGE2 EP4 expression in different cell lines. A-172 (A), T98G (B), U-138 MG (C), P1 (D) and P6 (E) cells were treated with celecoxib, 2,5-DMC and rofecoxib for 48 and 72 h. The concentration of compounds used in this assay provided at least 70% of cell viability. PGE2 EP4 and COX-2 protein levels were evaluated by western blot. DMSO was used as a treatment control. GAPDH was used as loading control. The closest protein standard marker band for respected analyzed protein is marked with expected size in kDa. Mostly the protein standard marker is added as separate picture taken. Black boxes represent the cropped bands taken for the analysis and presented in the main body of the manuscript. These were taken into densitometric analyses. [file 12885_2021_8164_MOESM1_ESM.pdf]
